# Global burden of self-harm and interpersonal violence and influencing factors study 1990–2019: analysis of the global burden of disease study

**DOI:** 10.1186/s12889-024-18151-3

**Published:** 2024-04-13

**Authors:** Xiaoding Zhou, Ruyu Li, Peixia Cheng, Xiaonan Wang, Qi Gao, Huiping Zhu

**Affiliations:** 1 Department of Epidemiology and Health Statistics, School of Public Health, Capital Medical University, Beijing, People’s Republic of China; 2 Beijing Municipal Key Laboratory of Clinical Epidemiology, Capital Medical University, Beijing, People’s Republic of China; 3 Department of Maternal and Child Health, School of Public Health, Capital Medical University, Beijing, People’s Republic of China; 4 Laboratory for Gene-Environment and Reproductive Health, Laboratory for Clinical Medicine, Capital Medical University, Beijing, People’s Republic of China

**Keywords:** Self-harm, Interpersonal violence, Influencing factors

## Abstract

**Introduction:**

Widespread concern exists in today’s world regarding self-harm and interpersonal violence. This study to analyze the changes in temporal trends and spatial patterns of risk factors and burdens of self-harm and interpersonal violence using the Global Burden of Diseases, Injuries, and Risk Factors Study (GBD) 2019.

**Methods:**

Temporal trends in self-harm and interpersonal violence were initially summarized using the estimated annual percentage change (EAPC). Data were compiled and visualized to delineate changes in disease burden and factors influencing self-harm and interpersonal violence from 1990 to 2019, stratified by gender, age and GBD region.

**Results:**

In 2019, the DALY rates of self-harm were 424.7(95% UI 383.25, 466.93). Over the period from 1999 to 2019, self-harm exhibited an overall decreasing trend, with the EAPC of -1.5351 (95% CI -1.6194, -1.4507), -2.0205 (95% CI -2.166, -1.8740) and -2.0605 (95% CI -2.2089, -1.9119), respectively. In contrast, the incidence rate of interpersonal violence was significantly higher than self-harm, with a rate of 413.44 (95% UI 329.88, 502.37) per 100,000 population. Mortality and DALYs of interpersonal violence were lower than those of self-harm, at 5.22 (95% UI 4.87, 5.63) and 342.43 (95% UI 316.61, 371.55). Disease burden of self-harm and interpersonal violence varied by gender, age groups and region. Specific risk factors showed that alcohol use, high temperature and drug use were the main risk factors for self-harm, while alcohol use, intimate partner violence and high temperature were associated with interpersonal violence. Low temperature was a common protective factor for both self-harm and interpersonal violence. The burden of self-harm and interpersonal violence was attributed to different factors influences in different SDI regions.

**Conclusions:**

The study explored temporal trends and spatial distribution of the global disease burden of self-harm and interpersonal violence, emphasizing the significant impact of factors such as alcohol use, temperature, and drug use on disease burden. Further research and policy actions are needed to interpret recent changes of disease burden of self-harm and interpersonal violence, and dedicated efforts should be implemented to devise evidence-based interventions and policies to curtail risk factors and protect high-risk groups.

**Supplementary Information:**

The online version contains supplementary material available at 10.1186/s12889-024-18151-3.

## Introduction

Intentional injuries, which included self-harm and interpersonal violence, were an important public health problem [1]. The high incidence and serious consequences of these behaviors place a heavy burden on individuals, families, and society. Approximately 817,000 people die by suicide each year worldwide, accounting for 2.2% of all deaths [2]. Global Burden of Disease (GBD) study estimated that 973 million people were injured and 4.8 million deaths from accidents and violence around the world in 2013. The leading causes of death were suicides and homicide. Among the people injured, 5.8% (56.2 million) had to be hospitalized, and 38.5% (21.7 million) sustained fractures [3].

Despite the growing recognition of the importance of self-harm and interpersonal violence, in-depth research on these issues remains relatively limited. Previous studies have documented the global prevalence of self-harm and interpersonal violence [4–7] however, they have focused mainly on particular populations and regions, and have not adequately addressed the burden of disease and the factors that influence self-harm or interpersonal violence. For instance, a review summarized the global prevalence of suicidal behavior, intentional self-harm, and non-suicidal self-harm, but only among young people [4]. Another study of the burden of self-harm in 2019 is only for young Europeans [8]. Similarly, Mercy’s article covers the global impact of interpersonal violence, but the analysis of trends in the burden of interpersonal violence and the factors that influence it is not very comprehensive [9]. A 2018 review summarizes the prevalence of interpersonal violence only in Latin America [10]. In addition, subgroup analyses and trend studies of the burden of self-harm and interpersonal violence are inadequate. An in-depth understanding of the global diversity of these issues and the mechanisms that influence them is essential.

Therefore, this study aims to provide insight into the global burden of self-harm and interpersonal violence. Through an exhaustive analysis of the GBD database, attention was paid to differences across gender, age groups, and regions to identify high-risk groups. Attributable influences on self-harm and interpersonal violence were also analyzed in depth to help identify key drivers that may influence the burden of self-harm and interpersonal violence. In addition, by calculating time trends from 1990–2019, we could capture trends in self-harm and interpersonal violence among different subgroups, as well as changes in influencing factors. With this study, we expect to support the global health field with more reliable data. Developing more effective health promotion programs to reduce the social burden of self-harm and interpersonal violence. We will make a lasting and positive contribution to building a safer and healthier global society.

## Methods

### Data sources

This study employed estimates from GBD 2019, which were available on the Global Health Data Exchange (GHDx) [11]. The GBD database provides comparable estimates of incidence, prevalence, mortality and DALYs for 369 diseases and injuries in 204 countries and territories between 1990 to and 2019. Data sources included all available data such as censuses, civil registration and vital statistics, disease registries, household surveys, surveillance, and verbal autopsies. Data and the protocol for the 2019 GBD can be accessed through the Global Health Data Exchange GBD Results Tool (http://ghdx.healthdata.org/gbd-results-tool) [4]. The GBD database’s studies complied with the Guidelines for Accurate and Transparent Health Estimates Reporting. This study focuses on the changes in disease burden and factors influencing self-harm and interpersonal violence across gender, age groups and 21 regions from 1990 to 2019.

### Risk factors

The GBD 2019 estimation of attributable burden followed the general framework established for comparative risk assessment (CRA) [5, 6] used since 2002. Details of the CRA methodology have been described in previous studies [7]. All risk factors contributing to the burden of self-injury and interpersonal violence available in the GBD 2019 database were captured in this study, including: 3 level-1 risk factors (behavioural risks, environmental and occupational risks, and metabolic risks), 5 level-2 risk factors (low bone mineral density, non-optimal temperature, tobacco, drug use, and alcohol use), and 3 level-3 risk factors (high temperature, low temperature, and smoking) [12]. If there was duplication of impact factors at different levels, the data for the lower level of impact factors would be retained.

### Definitions

#### Self-harm and interpersonal violence

Self-harm is a broad concept that encompasses degrees of intentionality that are hard to separate: from non-suicidal self-harm to attempted suicide to suicides [13, 14]. The World Health Organization (WHO) defines violence as the intentional use of power or force, threatened or actual, against oneself, another person, or a group or community, which either results in or has a likelihood of resulting in injury, death, psychological harm, dysplasia, or deprivation. Based on this definition, WHO separates violence into three broad groups, namely, self-directed violence, interpersonal violence, and collective violence. Interpersonal violence by itself divides into two categories, i.e., family and intimate partner violence (e.g., child abuse, violence by an intimate partner, and abuse of the elderly) and community violence (e.g., youth violence, rape or sexual assault by strangers, and violence in institutional settings) [15].

### DALYs

DALYs were defined as the sum of years lost due to premature death (YLLs) and years of healthy life lost due to disability (YLDs) [16]. DALYs were launched by the World Bank and are backed by the World Health Organization as a measure of the GBD. One DALY could be regarded as losing one year in full health [17].

### Socio-demographic Index (SDI)

The SDI used in this study was categorized into five groups: low SDI (0–0.45), low-middle SDI (0.45–0.61), middle SDI (0.61–0.69), high-middle SDI (0.69–0.81) and high SDI (0.81–1) [18]. SDI is a compound measure of income, average years of schooling, and fertility for each GBD location and year, which was initially constructed for GBD 2015 using the Human Development Index (HDI) methodology to measure Socio-demographic development.

### Analytic strategy

GBD 2019 estimation followed the methodology outlined in the previous published study [19]. Mortality estimates were calculated mainly using vital registration data or household mortality surveys, and statistical methods, such as noise reduction algorithms and Bayesian geospatial regression sofware, were used to enforce the comparability of mortality data sources. Incidence estimates was generated by using a broad range of population-representative data sources identified by literature review and via study collaborations. Epidemiologic state-transition disease modeling sofware, DisMod-MR, Bayesian meta-regression sofware, and MR-BRT were conducted to improve consistency between epidemiological parameters.

EAPC (age-standardized annual percentage change) [20] was conducted to measure the rate of change in incidence, mortality, and DALY rates from 1999 to 2019. Uncertainty intervals (UIs) were computed for all estimates at each step of the burden estimation process from the 2.5th and 97.5th powers of the 1000th sampling of the posterior distribution. A regression line was fitted to the natural logarithm of the rates, i.e.  , where  , and *x* = calendar year. The EAPC was calculated as  and its 95% confidence interval (CI) can also be obtained from the linear regression mode [21]. The rate was deemed to be an increasing trend when both the EAPC and the lower boundary of its 95%CI were greater than zero, and a decreasing trend was considered when both EAPC and the upper boundary of its 95%CI less than zero. Otherwise, the rate regarded as stable over time.

## Result

### Global burden of disease for self-harm and interpersonal violence

In 2019, the global incidence, mortality, and DALY rates per 100,000 population for self-harm and interpersonal violence combined were 614.44 (95% UI 510.28,720.97), 15.55 (95% UI 14.49,16.60), and 856.18 (95% UI 799.83,919.04), respectively. Of these, the DALY rate per 100,000 population for self-harm was 424.7(95% UI 383.25, 466.93). Overall incidence, mortality, and DALY rates for self-harm showed a decreasing trend from 1999–2019, with the EAPC of -1.5351 (95% CI -1.6194, -1.4507), -2.0205 (95% CI -2.166,-1.8740) and -2.0605 (95% CI -2.2089,-1.9119), respectively. And the incidence of interpersonal violence was substantially higher than self-harm in 2019, with a rate of 413.44 (95% UI 329.88, 502.37) and 62.48 (53.17, 73.88) per 100,000 population, respectively. Moreover, mortality and DALYs of interpersonal violence were lower than self-harm, at 5.22 (95% UI 4.87,5.63) and 342.43 (95% UI 316.61,371.55).

Table 1 differences were observed between self-harm and interpersonal violence disease burden in terms of gender, region, and age group. For self-harm, females were more likely to engage in self-injurious behavior than males, but males had higher rates of mortality and DALYs than females. Similarly, the EAPC for all three indicators showed a decreasing trend, but the rate of decline was slower for males than for females. The incidence of self-harm was highest in the high SDI across the five SDI regions and declined as the SDI declined. It was worth noting that there was an increase in low-middle SDI, and the low-middle SDI region had the highest rate of self-harm deaths and DALYs, with high SDI coming in second. High-middle SDI, middle SDI and low-middle SDI all showed a faster downward trend in EAPC than high SDI and low SDI. The incidence and DALYs rates of self-harm declined as age got older, and in 2019, young people aged 20–24 years had the highest incidence and DALYs rates of self-harm among all age groups, which were 133.44 (95% UI 93.00,187.68) and 781.20 (95% UI 706.85,871.71), respectively. And it decreased with increasing age. In contrast, the mortality rate of self-harm increased with the age, and the highest rate was 33.41(95% UI 28.1,37.22) in older people aged 85 years and older.

**Table 1:** Global incidence rate, mortality rate and DALYs rate for self-harm and interpersonal violence in 2019

	**Incidence rate**		**Mortality rate**		**DALYs**	
	**ASIR (95% UIs)**	**EAPC 1990–2019**	**ASMR (95% UIs)**	**EAPC 1990–2019**	**ASDR (95% UIs)**	**EAPC 1990–2019**
**Self-harm**						
Overall	62.48(53.17,73.88)	-1.5351 (-1.6194,-1.4507)	9.39(8.48,10.29)	-2.0205 (-2.1660,-1.8740)	424.70(383.25,466.93)	-2.0605(-2.2089,-1.9119)
Sex						
Male	50.99(43.63,59.91)	-0.9923 (-1.0898,-0.8946)	13.25(11.31,14.72)	-1.6133 (-1.7849,-1.4414)	576.96(492.88,643.38)	-1.5861 (-1.7491,-1.4228)
Female	74.04(62.64,87.59)	-1.8687 (-1.9557,-1.7817)	5.74(5.14,6.41)	-2.8261 (-2.9562,-2.6959)	273.72(244.66,306.32)	-2.9090 (-3.0721,-2.7457)
Region						
High SDI	117.14(102.05,135.50)	-0.0182 (-0.1498,0.1136)	11.18(10.72,11.63)	-0.7049 (-0.7867,-0.6231)	502.72(484.48,521.52)	-0.6859 (-0.7671,-0.6046)
High-middle SDI	66.94(57.24,78.74)	-1.8196 (-1.9400,-1.6990)	8.79(8.05,9.80)	-2.8349 (-3.1446,-2.5241)	395.33(363.12,437.42)	-2.7497 (-3.0600,-2.4384)
Middle SDI	47.24(39.79,56.18)	-2.1338 (-2.2684,-1.9991)	7.41(6.51,8.35)	-2.6597 (-2.7792,-2.5401)	317.88(281.11,356.08)	-2.7887 (-2.9214,-2.6558)
Low-middle SDI	63.44(52.27,76.81)	1.3743 (-1.4869,-1.2617)	11.99(10.22,13.65)	-1.5521 (-1.7185,-1.3855)	577.24(496.48,652.77)	-1.7408 (-1.9200,-1.5613)
Low SDI	36.74(31.27,43.10)	-0.6150 (-0.6790,-0.5511)	9.55(7.92,11.42)	-0.9348 (-1.0276,-0.8420)	384.98(320.56,464.31)	-1.0366 (-1.1267,-0.9463)
Age group (years)^a^						
0–9	0(0.00,0.00)	0(0.00,0.00)	0(0.00,0.00)	0(0.00,0.00)	0(0.00,0.00)	0(0.00,0.00)
10–14	33.96(19.37,55.67)	-1.2606 (-1.3536,-1.1676)	1.30(1.10,1.51)	-2.2567 (-2.4242,-2.0888)	99.89(85.00,116.08)	-2.2532 (-2.4198,-2.0863)
15–19	101.64(68.63,139.64)	-1.5401 (-1.6530,-1.4271)	7.02(6.32,7.86)	-2.2474 (-2.3620,-2.1328)	505.02(454.9,564.18)	-2.2469 (-2.3610,-2.1327)
20–24	133.44(93.00,187.68)	-1.6069 (-1.7038,-1.5099)	11.61(10.46,12.97)	-2.0665 (-2.2381,-1.8947)	781.20(706.85,871.71)	-2.0688 (-2.2393,-1.8981)
25–29	108.67(71.13,160.88)	-1.6823 (-1.7551,-1.6094)	12.06(10.91,13.40)	-1.9821 (-2.2126,-1.7511)	755.81(685.62,838.63)	-1.9854 (-2.2139,-1.7564)
30–34	89.57(59.59,132.90)	-1.7118 (-1.8025,-1.6211)	11.84(10.61,13.12)	-2.0321 (-2.2267,-1.8370)	685.67(618.97,758.50)	-2.0342 (-2.2261,-1.8420)
35–39	82.22(54.16,123.83)	-1.6496 (-1.7216,-1.5775)	12.14(10.85,13.31)	-2.0327 (-2.1685,-1.8967)	645.09(577.44,706.02)	-2.0317 (-2.1666,-1.8967)
40–44	76.72(50.90,111.21)	-1.5974 (-1.6846,-1.5101)	12.04(10.82,13.34)	-2.1715 (-2.3542,-1.9884)	582.17(524.23,643.11)	-2.1684 (-2.3492,-1.9872)
45–49	73.61(48.68,104.92)	1.4192 (-1.5920,-1.2461)	12.24(10.91,13.49)	-2.1236 (-2.3649,-1.8817)	532.67(477.57,585.25)	-2.1219 (-2.3593,-1.8839)
50–54	60.08(40.34,87.28)	-1.2856 (-1.4061,-1.1649)	13.08(11.76,14.52)	-2.1284 (-2.3067,-1.9498)	506.12(457.41,557.69)	-2.1225 (-2.2971,-1.9475)
55–59	54.89(38.66,75.60)	-1.1320 (-1.2122,-1.0518)	14.18(12.66,15.60)	-1.9413 (-2.0958,-1.7865)	482.01(433.28,528.61)	-1.9343 (-2.0866,-1.7818)
60–64	46.49(30.32,67.51)	-1.3845 (-1.5271,-1.2418)	14.49(12.95,15.85)	-1.9614 (-2.0787,-1.8440)	425.71(383.88,466.07)	-1.9479 (-2.0647,-1.8309)
65–69	43.17(29,60.37.00)	-1.5174 (-1.6698,-1.3648)	16.04(14.37,17.65)	-1.9798 (-2.1632,-1.7960)	396.71(358.87,434.25)	-1.9535 (-2.1348,-1.7718)
70–74	39.68(26.64,56.66)	-1.4756 (-1.6254,-1.3257)	19.38(17.12,21.49)	-2.0216 (-2.2133,-1.8295)	391.40(349.55,430.17)	-1.9855 (-2.1743,-1.7964)
75–79	45.58(30.94,65.33)	-1.2131 (-1.3715,-1.0545)	23.93(21.05,26.35)	-1.8069 (-1.9872,-1.6263)	382.32(337.76,419.17)	-1.7811 (-1.9551,-1.6068)
80–84	63.79(45.09,87.27)	-0.8219 (-0.9724,-0.6711)	29.99(26.22,33.35)	-1.7359 (-1.9065,-1.5651)	368.97(324.54,407.99)	-1.7173 (-1.8810,-1.5533)
85 +	59.01(44.94,76.50)	-1.5351 (-1.6194,-1.4507)	33.41(28.10,37.22)	-1.4956 (-1.6204,-1.3707)	294.32(252.29,326.46)	-1.5369 (-1.6531,-1.4205)
**Interpersonal violence**						
Overall	413.44(329.88,502.37)	-0.6175 (-0.6589,-0.5760)	5.22(4.87,5.63)	-1.4774 (-1.6542,-1.3003)	342.43(316.61,371.55)	-1.2818 (-1.4213,-1.1421)
Sex						
Male	610.77(488.29,741.30)	-0.5697 (-0.6147,-0.5247)	8.61(8.00,9.31)	-1.2857 (-1.4480,-1.1231)	528.74(490.89,571.90)	-1.1471 (-1.2823,-1.0116)
Female	212.92(168.41,260.10)	-0.7521 (-0.8059,-0.6983)	1.81(1.65,1.98)	-2.2725 (-2.5214,-2.0231)	153.20(135.65,173.54)	-1.7112 (-1.8744,-1.5478)
Region						
High SDI	472.23(357.59,587.53)	-0.5274 (-0.6275,-0.4272)	2.51(2.40,2.58)	-1.9196 (-2.1239,-1.7149)	188.61(176.69,202.20)	-1.6173 (-1.7989,-1.4354)
High-middle SDI	452.82(358.69,548.37)	-0.9684 (-1.0225,-0.9144)	3.97(3.72,4.25)	-3.4016 (-4.0010,-2.7985)	265.74(246.68,287.05)	-2.9723 (-3.4534,-2.4888)
Middle SDI	402.27(317.54,489.80)	-0.2940 (-0.3366,-0.2514)	5.59(5.15,6.10)	-1.7419 (-1.8984,-1.5852)	362.60(334.67,393.47)	-1.5879 (-1.7320,-1.4437)
Low-middle SDI	448.40(362.94,539.91)	-0.5396 (-0.5993,-0.4798)	6.48(5.90,7.17)	-0.2209 (-0.3776,-0.0639)	407.59(371.00,447.49)	-0.2007 (-0.3443,-0.0569)
Low SDI	345.24(280.23,413.50)	-0.1838 (-0.2170,-0.1505)	7.13(5.71,8.43)	-0.5790 (-0.7021,-0.4557)	424.95(348.60,498.91)	-0.4781 (-0.5904,-0.3657)
Age group (years)^a^						
< 5	203.07(131.21,291.26)	-0.9827 (-1.1484,-0.8167)	1.86(1.41,2.35)	-2.1999 (-2.3254,-2.0743)	165.34(126.57,208.40)	-2.1871 (-2.3122,-2.0620)
5–9	185.92(112.83,288.26)	-0.3838 (-0.6064,-0.1607)	0.76(0.61,0.92)	-2.2003 (-2.3360,-2.0645)	72.72(60.08,85.77)	-2.0097 (-2.1308,-1.8885)
10–14	317.59(179.78,529.10)	-0.3941 (-0.5403,-0.2476)	1.08(0.92,1.24)	-1.2251 (-1.3512,-1.0988)	103.78(89.54,118.54)	-1.0959 (-1.1968,-0.9949)
15–19	610.12(352.61,935.14)	-0.6671 (-0.7192,-0.6150)	5.96(5.39,6.59)	-0.7998 (-0.9847,-0.6145)	469.68(424.95,518.91)	-0.7753 (-0.9402,-0.6101)
20–24	786.21(493.06,1126.32)	-0.7073 (-0.8092,-0.6053)	9.84(9.11,10.67)	-0.9076 (-1.1282,-0.6865)	722.59(667.25,789.79)	-0.8699 (-1.0693,-0.6702)
25–29	726.75(458.43,1049.46)	-0.6456 (-0.7161,-0.5750)	9.32(8.68,10.07)	-1.1257 (-1.3050,-0.9460)	653.51(604.28,708.94)	-1.0550 (-1.2157,-0.8940)
30–34	610.42(382.00,952.38)	-0.7324 (-0.8038,-0.6609)	8.63(8.05,9.26)	-1.3874 (-1.5004,-1.2743)	572.42(529.18,620.20)	-1.2819 (-1.3817,-1.1819)
35–39	513.51(320.18,778.46)	-0.7597 (-0.8272,-0.6923)	8.14(7.58,8.77)	-1.5899 (-1.8153,-1.3640)	506.15(466.76,549.44)	-1.4409 (-1.6352,-1.2462)
40–44	437.10(256.93,672.75)	-0.6535 (-0.7063,-0.6007)	7.16(6.64,7.72)	-1.8987 (-2.1959,-1.6006)	422.57(386.85,462.17)	-1.6629 (-1.9109,-1.4143)
45–49	382.33(229.16,596.18)	-0.4145 (-0.5029,-0.3260)	5.94(5.53,6.45)	-2.0122 (-2.3346,-1.6887)	337.38(307.96,374.00)	-1.7034 (-1.9629,-1.4432)
50–54	319.97(201.37,463.23)	-0.2880 (-0.3726,-0.2033)	5.19(4.83,5.59)	-1.9379 (-2.2155,-1.6594)	276.39(250.76,306.11)	-1.6221 (-1.8344,-1.4094)
55–59	255.63(160.72,388.90)	-0.2867 (-0.3238,-0.2495)	4.78(4.46,5.14)	-1.8948 (-2.2018,-1.5868)	232.69(209.96,258.90)	-1.5535 (-1.7803,-1.3263)
60–64	211.26(122.30,321.27)	-0.4114 (-0.4783,-0.3445)	4.24(3.97,4.55)	-1.9740 (-2.2997,-1.6472)	193.56(172.42,217.87)	-1.5239 (-1.7480,-1.2994)
65–69	188.17(111.24,289.25)	-0.3301 (-0.4031,-0.2571)	3.87(3.63,4.12)	-1.8108 (-2.1253,-1.4954)	164.92 (145.45,188.69)	-1.3143 (-1.5174,-1.1108)
70–74	187.65(115.81,280.62)	-0.2144 (-0.2696,-0.1592)	3.86(3.62,4.13)	-1.5688 (-1.8392,-1.2977)	145.87 (126.89,168.36)	-1.0777 (-1.2409,-0.9142)
75–79	174.49(109.32,259.53)	-0.2578 (-0.3057,-0.2099)	3.94(3.64,4.22)	-1.6007 (-1.8285,-1.3724)	128.13 (110.56,149.80)	-1.0327 (-1.1544,-0.9109)
80–84	163.15(100.20,261.52)	-0.5015 (-0.5778,-0.4252)	2.07(1.85,2.24)	-2.4189 (-2.6559,-2.1814)	88.72 (72.72,109.15)	-1.0748 (-1.1575,-0.9921)
85 +	208.31(140.58,305.50)	-0.6029 (-0.6593,-0.5465)	2.17(1.81,2.38)	-2.3634 (-2.5885,-2.1377)	74.37 (59.08,94.23)	-1.1188 (-1.1989,-1.0387)

*ASIR* Age-standardised incidence rates (per 100,000 population), *ASMR* Age-standardised mortality rates (per 100,000 population), *ASDR* Age-standardised DALYs rates (per 100,000 population), *UIs* Uncertainty IntervalIs

Age group (years)^a^unadjusted rates (per 100,000 population)

The distribution and trends of interpersonal violence were different from those of self-harm. Males had higher rates of incidence, mortality, and DALYs of interpersonal violence than females. The EAPC for all three indicators showed a decreasing trend and decreased more slowly for males than females. The incidence of interpersonal violence was highest in high SDI across the five SDI regions, and as the SDI declined, the incidence of interpersonal violence declined with it. And similar to self-harm, there was an increase in Low-middle SDI. Whereas mortality and DALY rates had been consistently trending downward with decreasing SDI. In 2019, people aged 20–24 years had highest rates of interpersonal violence, mortality, and DALYs were 786.21 (95% UI 493.06,1126.32), 9.84 (95% UI 9.11,10.67), and 722.59 (95% UI 667.25,789.79), respectively. Trends over time were similar in all three indicators of self-harm and interpersonal violence, with an overall downward trend from 1999–2019.

### Attributable burden by impact factor

**Fig. 1:**
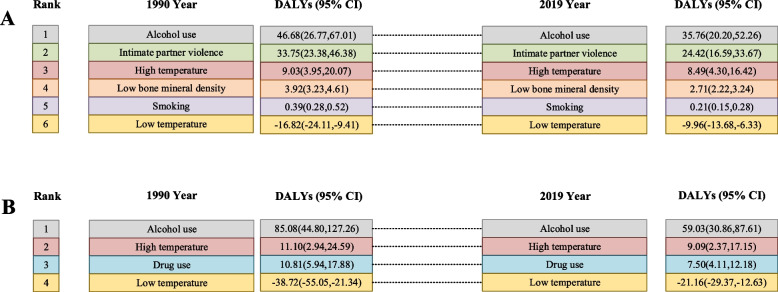
Changes in the ranked contribution of risk factors to the global burden of disease for self-harm and interpersonal violence between 1990 and 2019. **A** Changes in the ranked contribution of risk factors to the global burden of disease for interpersonal violence between 1990 and 2019. **B** Changes in the ranked contribution of risk factors to the global burden of disease for self-harm between 1990 and 2019

Figure 1 showed the ranking of the contribution of each risk factor to the global burden of disease for self-harm and interpersonal violence DALYs in 1999 and 2019. There were four categories of risk factors for self-harm DALYs, including alcohol use, high temperature and drug use. They were ranked first, second, and third in that order. Low temperature was the protective factor. The leading five risk factors in terms of attributable interpersonal violence DALYs were alcohol use, intimate partner violence, high temperature, low bone mineral density, and smoking. Low temperature was considered a protective factor for interpersonal violence DALYs. Ranking of risk factors by attributable self-harm and interpersonal violence DALYs globally remained unchanged between 1990 and 2019.

**Fig. 2:**
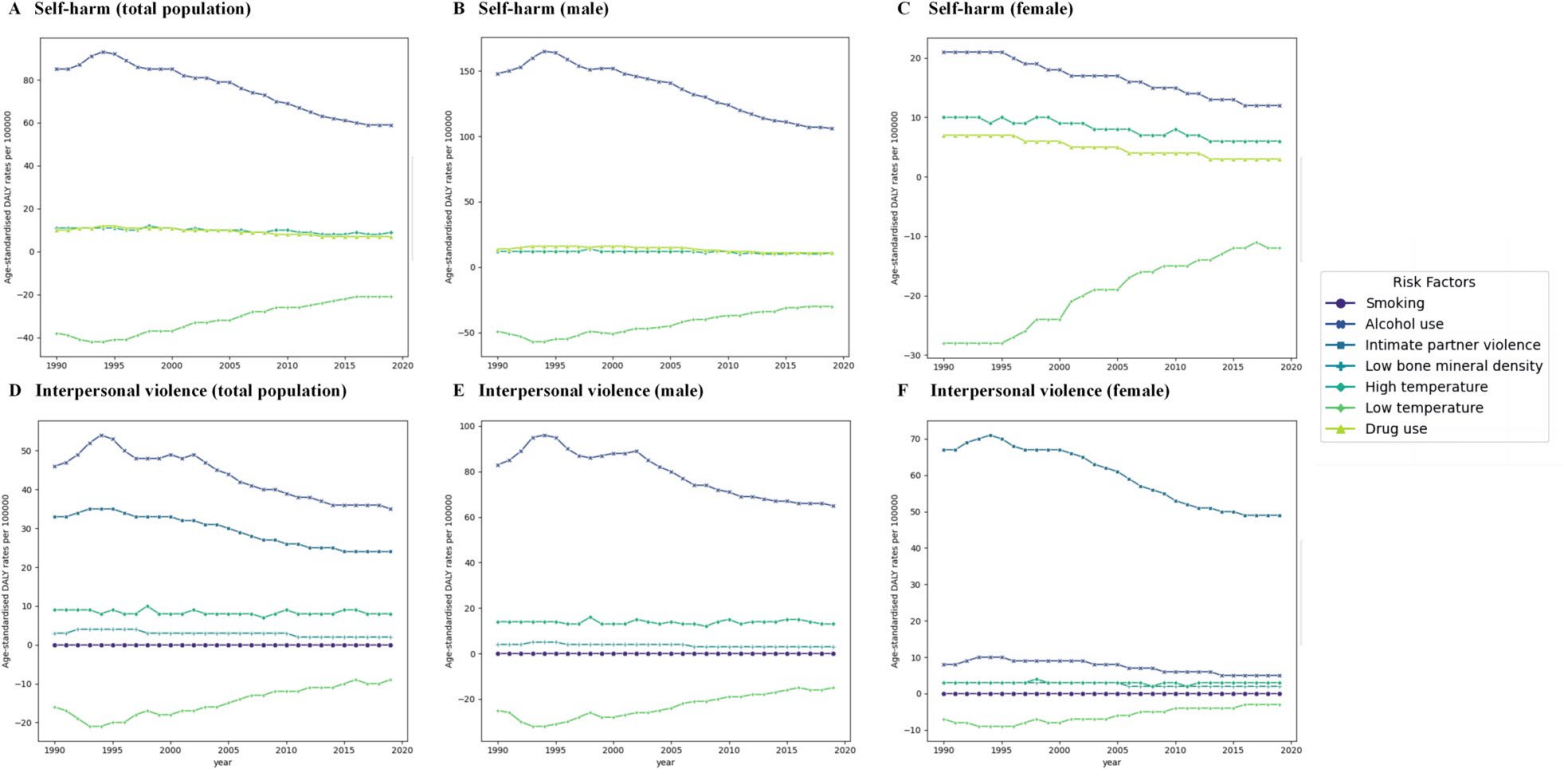
Age-standardised DALY rates for self-harm and interpersonal violence per 100,000 population by sex, 1990–2019. **A**-**C** Self-harm ASDR. **D**-**F** Interpersonal violence ASDR. ASDR = Age-standardised DALY rates

During 1999–2019, alcohol use was the primary risk factor in terms of risk-attributable self-harm Age-standardised DALY rates (ASDR) globally for both males and females, and the self-harm ASDR attributable to all risk factors was 6–8 times higher in males than in females (Fig. 2). Temporal trends in attributable self-harm ASDR due to drug use and high temperatures were generally consistent, but attributable self-harm ASDR for drug use was 1–3 times lower in females than in males (Tables S1, and S2). Alcohol use was the leading risk factors in terms of attributable interpersonal violence ASDR, followed by intimate partner violence, high temperature, low bone mineral density, and smoking. Subgroup analysis by gender displayed that intimate partner violence was not a risk factor influencing interpersonal violence in males, and the other four risk factors were generally consistent with the total population. Whereas among females, the pattern was different. Intimate partner violence was the paramount risk factor in terms of attributable interpersonal violence ASDR in females, and the ASDR attributable to alcohol use was dramatically lower than in males. In addition, low hypothermia was a protective factor for both self-harm and interpersonal violence ASDR, but the protective effect of low hypothermia diminished gradually over time.

**Fig. 3:**
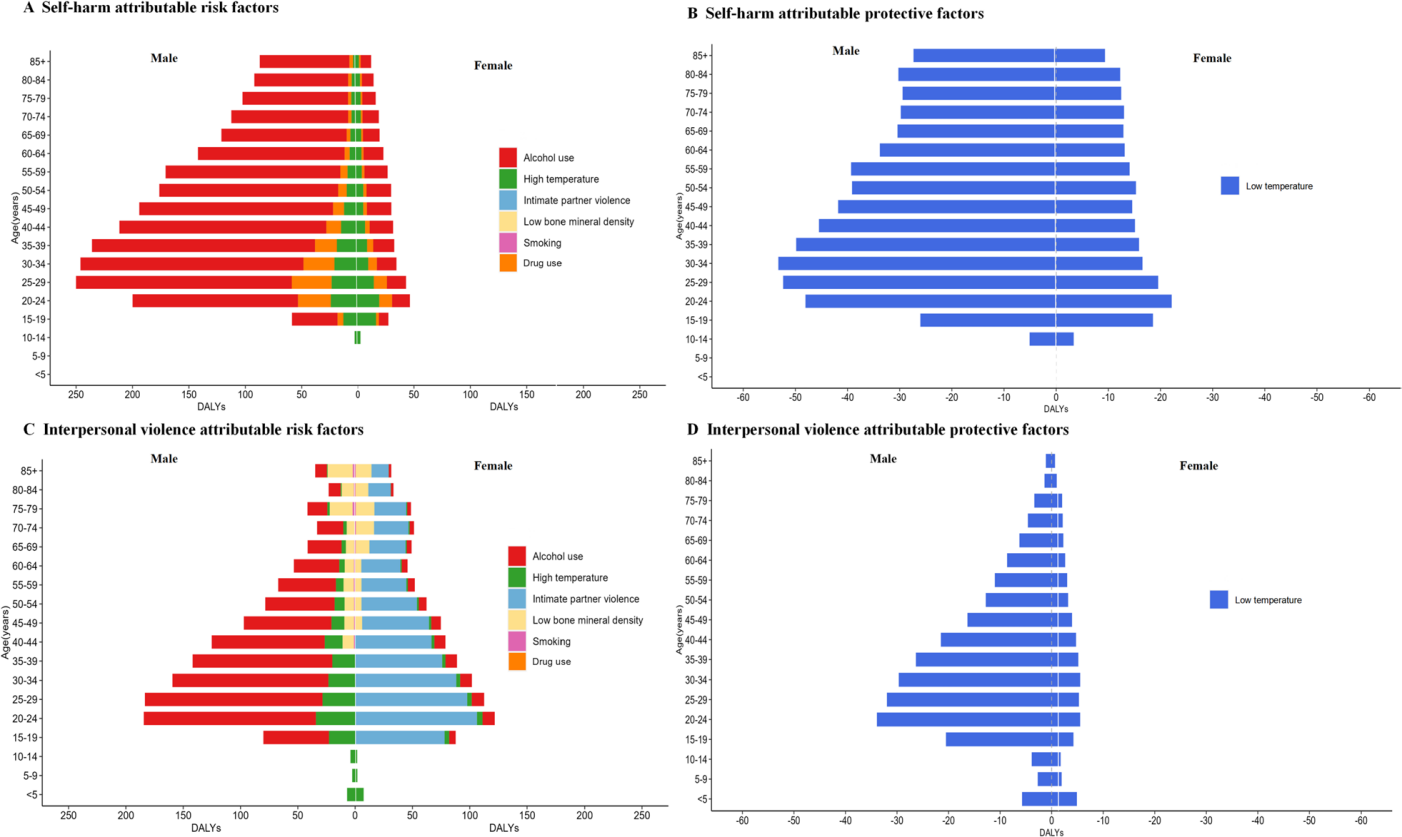
Global DALYs for self-harm and interpersonal violence attributable to impact factors by type, sex, and age, 2019. **A** Self-harm attributable risk factors. **B** Self-harm attributable protective factors. **C** Interpersonal violence attributable risk factors. **D** Interpersonal violence attributable protective factors

Figure 3 showed estimates of attributable DALY rates for self-harm and interpersonal violence for different age and gender groups in 2019. Alcohol use was the main risk factor for the burden of self-harm among those aged 20 years and older, especially among men, although this effect declined slightly with age. The rate of attributable DALYs was higher for drug use among those aged 20–40 years. Rates of attributable DALYs for interpersonal violence showed significant gender differences. Alcohol use and intimate partner violence were major risk factors in the age group over 15 years, especially in the 20–30 age group. ASDR attributable to alcohol use was much lower in females than in males. In addition, we found that low bone mineral density appeared earlier and was heavier in males, especially in older adults over the age of 85 years. DALYs attributable to temperature was lower in women than men, both as a risk factor for high temperature and as a protective effect of low temperature.

**Fig. 4:**
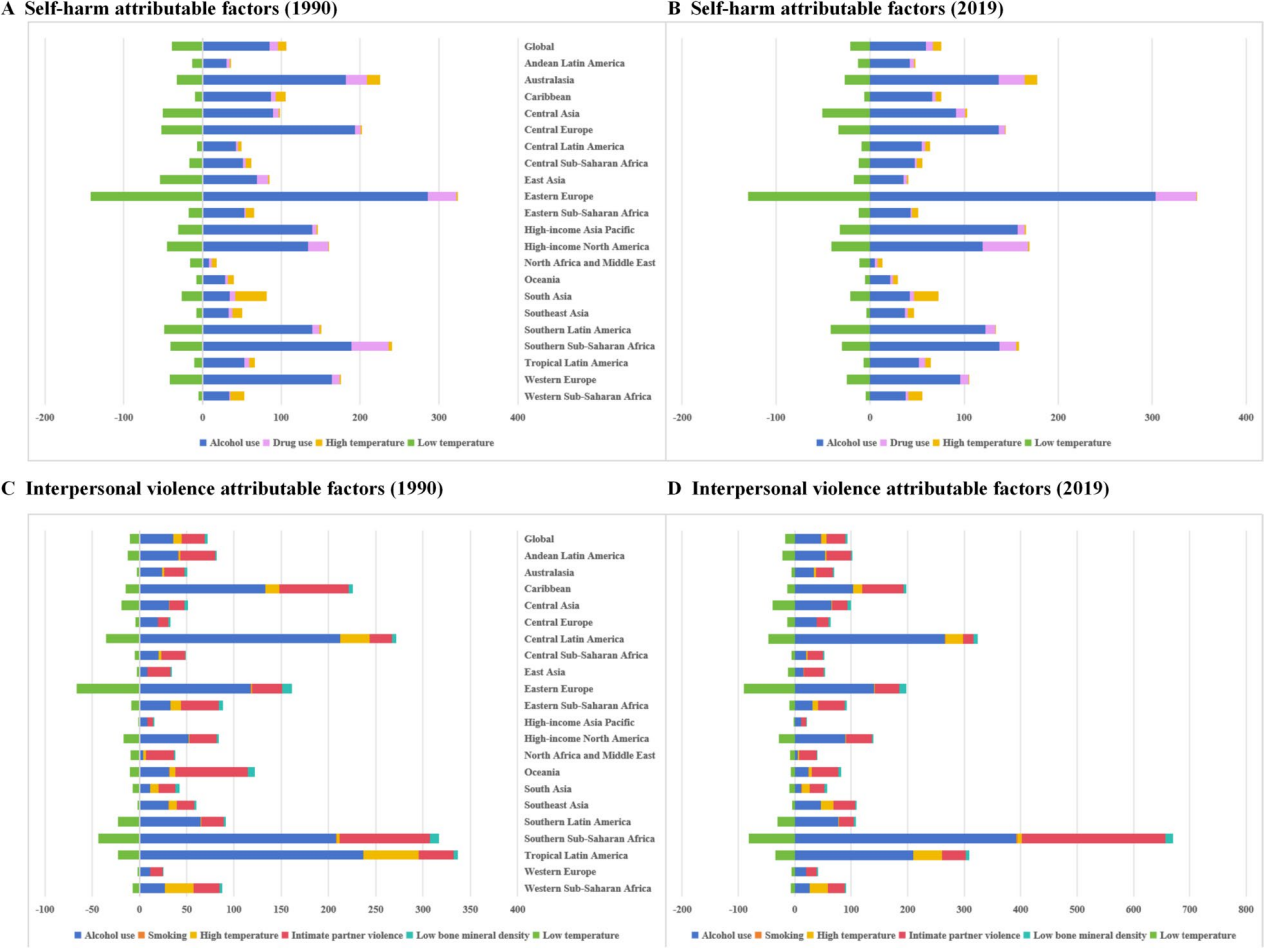
Global ASDR for self-harm and interpersonal violence attributable impact factors by SDI regions. **A** Self-harm attributable factors (1990). **B** Self-harm attributable factors (2019). **C** Interpersonal violence attributable factors (1990). **D** Interpersonal violence attributable factors (2019)

Geographical patterns for self-harm and interpersonal violence DALYs attributable to influencing factors in 2019 differed around the world (Fig. 4), with the highest self-harm DALYs attributable to alcohol use and drug use in Eastern Europe, high-income Asia Pacific, and high-income North America (high SDI level regions). Regions with the highest self-harm DALYs attributable to alcohol use and high temperature were those with medium and low SDI levels, such as South Asia, Australasia, and Western Sub-Saharan Africa. However, the pattern of interpersonal violence was extremely different from self-harm. Interpersonal violence ASDR attributable to alcohol use, intimate partner violence and high temperature were most severe in regions with medium SDI levels, including Central Latin America, Tropical Latin America, and the Caribbean. Additionally, interpersonal violence ASDR attributable to alcohol use, intimate partner violence, and low bone mineral density were more prevalent and was found in high, medium, and low SDI regions, most severely in Southern Sub-Saharan Africa, Oceania and Eastern Europe. In the Eastern Europe region, low temperatures had a significant effect on both self-harm and interpersonal violence. In terms of temporal trend, self-harm and interpersonal violence attributable to influencing factors were generally consistent in 1990 and 2019.

## Discussion

Globally, the strength of this study was the comprehensive description of the burden of disease for self-harm and interpersonal violence. Additionally, this study analyzed their temporal and spatial trends, gender-age differences, and associated risk factors. From 1990 to 2019, the global burden of self-harm and interpersonal violence showed a decreasing trend. However, the prevalence of interpersonal violence remains significantly higher than self-harm. Subgroup analyses revealed that females were more likely to self-harm, while males were more likely to experience interpersonal violence, and that the occurrence of self-harm and interpersonal violence was more pronounced in the 20–24 year age group and in high SDI areas. Major risk factors included alcohol consumption and high temperatures. Notably, low temperatures was a common protective factor for both self-harm and interpersonal violence. This study provides the basis for national or regional development of targeted and effective interventions to improve the health and well-being of populations.

### Burden of self-harm and interpersonal violence

Our study showed that the incidence of interpersonal violence was significantly higher than that of self-harm. This difference might stem from the more visible and easily documented nature of interpersonal violence in society. Much of the interpersonal violence occurred at work, in public places, and social settings [22], making them more noticeable. In addition, widespread public condemnation of interpersonal violence might further motivate people to report and expose these behaviours, thus increasing reporting rates [23]. In contrast, self-harm behaviours might be more likely unreported because of social discrimination and individual privacy [24].

Although the incidence of self-harm in our study was somewhat lower compared to interpersonal violence, it is worth noting that self-harm is one of the most common reasons for emergency department visits and admissions, and significantly increases the risk of future suicide deaths [25], which means that more intervention is still needed. In addition, previous studies have confirmed that mental health problems might be one of the core causes of serious consequences of self-harm [26]. Therefore, society needed to focus on mental health promotion and outreach to increase awareness of this area and strengthen support systems to mitigate the negative impact of self-injurious behaviour on patients and society. Encouragingly, the global incidence, mortality, and DALYs of self-harm and interpersonal violence had been trending downwards over the past 20 years (1990–2019). This reflected increased global awareness of mental health issues and improvements in related interventions. Society was gradually recognizing the critical role of mental health in overall health, enhancing access to mental health services and social support systems [27–29]. These positive efforts might have improved individual mental health, thereby contributing to a reduction in the prevalence of self-harm and the associated burden of death and disease. In summary, our study highlighted differences in the prevalence and degree of impact of interpersonal violence and self-injurious behaviour. This not only provided direction for future in-depth research but also highlighted the need for society to recognize mental health issues more comprehensively and adopt more targeted measures in prevention and intervention.

### Factors influencing self-harm and interpersonal violence

We observed that alcohol use was the most crucial risk factor for both self-harm and interpersonal violence. A prevailing hypothesis for these patterns suggests that alcohol use may affect an individual’s neurological and psychological state [30], weakening inhibitions and judgment, and prompting more impulsive, risky, and aggressive behaviours [31], which may make people more susceptible to suffer head injuries and concussions while under the influence of alcohol [32]. Furthermore, we also found that drugs use, like alcohol use, acted as a key risk factor contributing to the global burden of self-harm and interpersonal violence. This might be closely related to the physiological and psychological effects it triggers [33, 34]. Therefore, reducing the frequency and amount of alcohol and drug use was one of the effective measures to prevent self-harm and interpersonal violence.

In our study, high temperatures had been cited as a risk factor for self-harm and interpersonal violence, whereas low temperatures had been seen as a protective factor. This might be related to the effect of temperature on an individual's psychological state and behaviour [35]. High temperatures might lead to physical discomfort and fatigue, which could exacerbate mood swings and psychological stress, which in turn increased the risk of self-harm and interpersonal violence [36]. Secondly, ambient temperatures could affect people's outdoor activities and social contact opportunities, increasing interpersonal conflict or creating a suitable environment for crime [37]. Conversely, moderately low temperatures might contribute to individual comfort and psychological well-being, reducing the burden of both [38–40]. Low bone mineral density occupied a key position in the ranking as one of the risk factors for interpersonal violence, which may be related to the fact that people are physically weaker and more vulnerability to injury in a low bone mineral density state [41, 42]. It could also be influenced by social perceptions and aesthetics, making it more likely to be a victim of interpersonal violence.

Another intriguing phenomenon was that the ranking of the burden of disease for self-inflicted injuries and interpersonal violence due to risk factors did not change between 1990 and 2019. This suggested that although we had made some progress over the past decades, relevant interventions might lag. However, the relatively stable level of influence of factors on self-harm and interpersonal violence suggested the need for continued in-depth research and effective interventions on risk factors such as alcohol use, intimate partner violence, and drugs.

### Analysis of self-harm and interpersonal violence by sex, age, and region

Our study unveiled notable gender disparities in self-harm through subgroup analysis of men and women, indicating a higher prevalence among females in engaging in self-injurious behaviors compared to their male counterparts. Intriguingly, despite the higher incidence among females, males exhibited elevated rates of mortality and DALYs, aligning with established literature [43]. Women exhibited a twofold higher likelihood of experiencing depression compared to men, with a heightened prevalence of affective disorders among the female population [44]. This finding could elucidate that women were more prone than men to contemplate and engage in self-injurious behavior [45]. And the results on interpersonal violence were in line with the findings of a US study. Compared to women, men were more likely to experience any violence, single-experience and recurrent violence, physical attack, threat by weapon, violence in adolescence and adulthood, and violence perpetrated by friends, acquaintances, and strangers [46].

This study also showed that males had significantly higher self-harm and interpersonal violence DALYs attributable to alcohol use, drug use, and temperature than females. Compared to females, males might tend to choose more lethal means of self-harm and interpersonal violence [47, 48], and were more reluctant to seek help after injury. They were less likely to take appropriate wound management measures, which may lead to more serious medical complications [44, 49]. According to the results of this study, intimate partner violence was the most significant risk factor for interpersonal violence in women and is much higher than in men [50]. Intimate partner violence was a serious social and public health problem that not only caused physical and psychological harm to victims but also increased the global burden of disease from interpersonal violence [51]. This might be related to factors such as women’s vulnerability in the family and society, exposure to gender discrimination, and culture of violence [52, 53]. Therefore, improving the social status and rights of women is an extremely important way to protect women from interpersonal violence. In addition, it was worth noting that although women are more sensitive to hot and cold environments due to physical and psychological dimensions [54]. However, temperature has a significantly greater impact on the burden of self-harm and interpersonal violence in males than in females.

We observed a heightened prevalence of self-harm and interpersonal violence in the 20–24 age group. With the transition to emerging adulthood [55], this period was seen as transformation. Although violent behaviour usually decreases with age [56], it did not entirely disappear in adulthood. During adolescence and emerging adulthood, individuals’ self-awareness and decision-making skills are not fully developed [57], and they are more susceptible to peer influence [58] and have difficulty in making rational decisions, which may increase the likelihood of involvement with alcohol, drugs and violence. This stage was also accompanied by changes in educational and occupational choices, as well as the reshaping of social relationships, leading to different forms of stress and anxiety. In this context, alcohol and drug use might be seen as a way of coping with these changes [59]. Simultaneously, the older population (≥ 85 years) experienced an elevated rate of mortality due to self-harm, potentially associated with aging factors and complications arising after acts of self-harm [60]. These findings underscored the imperative to implement targeted interventions tailored to different age groups, intending to effectively diminish the occurrence of self-harm and interpersonal violence.

In addition, it was found that the prevalence of self-harm and interpersonal violence declined with decreasing SDI, with higher detection and reporting rates in high SDI areas, which were economically and medically advanced and faced more intense social competition and more pronounced substance abuse problems [61]. However, it was worth noting that the trend of higher prevalence of self-harm in low-middle SDI areas might stem from the high stigma attached to mental health problems in the political culture of some countries, which led to individuals’ reluctance to seek medical help, exacerbated the burden of physical and mental health, and created unfavourable conditions for increased self-injurious behaviours [62]. Some studies pointed to the highest prevalence of daily alcohol abuse in the European region and the highest rates of dependence on drugs such as cannabis, opioids, and cocaine in the high-income regions of North America [63]. Some countries in the European region had a long history of wine-making and were more prone to self-harm behaviours because of their traditional culture and higher social tolerance of alcohol. High temperature was noteworthy among the factors contributing to the burden of self-inflicted disease in low-middle SDI regions such as South Asia, Australasia, and Western Sub-Saharan Africa. In Central Latin America, Tropical Latin America, and Caribbean, high temperature and intimate partner violence were also noteworthy. These areas were located near the equator, where the climate was hot, summer temperatures were higher, and the frequency and intensity of hot weather could be more significant. Prolonged exposure to high temperatures had a psycho-emotional impact and increased the likelihood of behaviours such as self-harm and interpersonal violence [64]. In addition, low levels of education and more traditional values, as well as attitudes towards family and gender roles, might account for the high prevalence of interpersonal violence in these areas, which was consistent with most studies [65, 66]. It was worth noting that for both self-harm and interpersonal violence, low temperatures had a significant effect in the Eastern Europe region. Some research suggested that exposure to cold water might help relieve stress and anxiety and promote the release of beneficial hormones in the body [67]. Longer winters in Eastern Europe might make it easier for people to experience feelings of exhilaration and reduce the incidence of self-harm and interpersonal violence. There were various reasons for differences between countries and regions, including SDI levels, different habits, folklore, natural ecosystems, demographic characteristics, national policies, and national health awareness.

### Limitations

There were some limitations of this study that were worth noting. Firstly, whilst we used the latest estimates data provided by GBD study, the restrictions of the GBD data would affect our findings. For instance, the limited temporal scope (1990 to 2019) of our research underscored the need for careful consideration when generalizing findings to other time periods. Similarly, the insufficient data availability in the GBD database hindered the comprehensive analysis of several common risk factors (e.g., mental health, family environment, and childhood experiences). Thirdly, we oversimplified the complexity of socio-demographic development by categorizing regions into different SDI classifications, and focusing on these regional classifications rather than individual countries might mask inter-country variability. Lastly, observational data make it difficult to obtain causality. Future cohort studies with individual-level data are recommended to validate these associations.

## Conclusion

Over the past two decades (1990–2019), there has been an encouraging downward trend in the global incidence of self-harm and interpersonal violence, as well as in mortality and disability-adjusted life years (DALYs). Despite the overall reduction in self-harm and interpersonal violence, the prevalence of interpersonal violence remained significantly higher than self-harm. Subgroup analyses by gender, age groups, and region revealed that females had a higher incidence of self-harm behaviors and males were more susceptible to interpersonal violence, especially in the 20–24 year age group. Additionally, the incidence of self-harm and interpersonal violence was most pronounced in high SDI regions. Major risk factors for self-harm and interpersonal violence globally included alcohol use and high temperatures. Further research and policy actions are needed to interpret recent changes of disease burden of self-harm and interpersonal violence, and dedicated efforts should be implemented to devise evidence-based interventions and policies to curtail risk factors and protect high-risk groups.

### Supplementary Information

**Supplementary Material 1.**

## Data Availability

The datasets generated and analyzed during the current study are available in the Global Burden of Diseases, Injuries, and Risk Factors Study (GBD) 2019, https://vizhub.healthdata.org/gbd-results/ (accessed on 1 Oct 2023).
