# Supplementary material for: Global burden of self-harm and interpersonal violence and influencing factors study 1990–2019: analysis of the global burden of disease study
Source: BMC Public Health. 2024 Apr 13;24:1035. doi: 10.1186/s12889-024-18151-3 (PMC11016221; doi:10.1186/s12889-024-18151-3)
Supplement: Supplementary file 1 — Supplementary Material 1. [file 12889_2024_18151_MOESM1_ESM.doc]

| Table S1 Self-harm drinking factors by gender, year. | | | |
| --- | --- | --- | --- |
| Year | Male | Female | Male-to-female Ratio |
| 1990 | 148.85 | 21.58 | 6.90 |
| 1991 | 150.35 | 21.25 | 7.08 |
| 1992 | 153.87 | 21.09 | 7.30 |
| 1993 | 160.97 | 21.22 | 7.58 |
| 1994 | 165.77 | 21.39 | 7.75 |
| 1995 | 164.32 | 21.05 | 7.81 |
| 1996 | 159.13 | 20.26 | 7.86 |
| 1997 | 154.41 | 19.67 | 7.85 |
| 1998 | 151.91 | 19.23 | 7.90 |
| 1999 | 152.95 | 19.00 | 8.05 |
| 2000 | 152.22 | 18.54 | 8.21 |
| 2001 | 148.25 | 17.98 | 8.25 |
| 2002 | 146.14 | 17.68 | 8.27 |
| 2003 | 144.83 | 17.62 | 8.22 |
| 2004 | 142.35 | 17.33 | 8.21 |
| 2005 | 141.55 | 17.19 | 8.23 |
| 2006 | 136.31 | 16.57 | 8.22 |
| 2007 | 132.71 | 16.18 | 8.20 |
| 2008 | 130.63 | 15.94 | 8.20 |
| 2009 | 126.43 | 15.53 | 8.14 |
| 2010 | 124.11 | 15.12 | 8.21 |
| 2011 | 120.44 | 14.64 | 8.23 |
| 2012 | 117.53 | 14.08 | 8.35 |
| 2013 | 114.74 | 13.66 | 8.40 |
| 2014 | 112.23 | 13.31 | 8.44 |
| 2015 | 111.03 | 13.09 | 8.48 |
| 2016 | 109.27 | 12.79 | 8.55 |
| 2017 | 107.45 | 12.44 | 8.63 |
| 2018 | 107.35 | 12.26 | 8.75 |
| 2019 | 106.31 | 12.04 | 8.83 |

| Table S2 Self-harm drug use factors by gender, year. | | | |
| --- | --- | --- | --- |
| Year | Male | Female | Male-to-female Ratio |
| 1990 | 14.20 | 7.36 | 1.93 |
| 1991 | 14.55 | 7.27 | 2.00 |
| 1992 | 15.17 | 7.25 | 2.09 |
| 1993 | 16.19 | 7.05 | 2.29 |
| 1994 | 16.93 | 7.38 | 2.29 |
| 1995 | 16.96 | 7.45 | 2.28 |
| 1996 | 16.53 | 7.13 | 2.32 |
| 1997 | 16.12 | 6.93 | 2.33 |
| 1998 | 15.96 | 6.84 | 2.33 |
| 1999 | 16.30 | 6.61 | 2.46 |
| 2000 | 16.46 | 6.28 | 2.62 |
| 2001 | 16.04 | 5.89 | 2.72 |
| 2002 | 15.83 | 5.52 | 2.87 |
| 2003 | 15.79 | 5.31 | 2.97 |
| 2004 | 15.64 | 5.26 | 2.98 |
| 2005 | 15.69 | 5.16 | 3.04 |
| 2006 | 15.02 | 4.93 | 3.05 |
| 2007 | 14.38 | 4.65 | 3.09 |
| 2008 | 13.91 | 4.47 | 3.11 |
| 2009 | 13.24 | 4.33 | 3.06 |
| 2010 | 12.89 | 4.27 | 3.02 |
| 2011 | 12.43 | 4.17 | 2.98 |
| 2012 | 12.10 | 4.01 | 3.02 |
| 2013 | 11.88 | 3.87 | 3.07 |
| 2014 | 11.79 | 3.73 | 3.16 |
| 2015 | 11.80 | 3.68 | 3.21 |
| 2016 | 11.76 | 3.76 | 3.13 |
| 2017 | 11.54 | 3.82 | 3.02 |
| 2018 | 11.36 | 3.85 | 2.95 |
| 2019 | 11.17 | 3.79 | 2.95 |
